# Multifractal Analysis and Experimental Evaluation of MCM-48 Mesoporous Silica as a Drug Delivery System for Metformin Hydrochloride

**DOI:** 10.3390/biomedicines12122838

**Published:** 2024-12-13

**Authors:** Mousa Sha’at, Maria Ignat, Liviu Sacarescu, Adrian Florin Spac, Alexandra Barsan (Bujor), Vlad Ghizdovat, Emanuel Nazaretian, Catalin Dumitras, Maricel Agop, Cristina Marcela Rusu, Lacramioara Ochiuz

**Affiliations:** 1 Department of Pharmaceutical Technology, Faculty of Pharmacy, ”Grigore T. Popa” University of Medicine and Pharmacy Iasi, 700115 Iasi, Romania; mousa-shaat@umfiasi.ro (M.S.); alexandra.m.bujor@umfiasi.ro (A.B.); lacramioara.ochiuz@umfiasi.ro (L.O.); 2 Laboratory of Material Chemistry, Department of Chemistry, ”Alexandru Ioan Cuza” University of Iasi, Bv. Carol I, no. 11, 700506 Iasi, Romania; maria.ignat@uaic.ro; 3 Department of Inorganic Polymers, Petru Poni Institute of Macromolecular Chemistry, 41A Grigore Ghica Voda Alley, 700487 Iasi, Romania; livius@icmpp.ro; 4 Department of Physical Chemistry, Faculty of Pharmacy, ”Grigore T. Popa” University of Medicine and Pharmacy Iasi, 700115 Iasi, Romania; adrian.spac@umfiasi.ro; 5 Biophysics and Medical Physics Department, “Grigore T. Popa” University of Medicine and Pharmacy Iasi, 700115 Iasi, Romania; 6 Faculty of Machine Manufacturing and Industrial Management, “Gheorghe Asachi” Technical University, 700050 Iasi, Romania; nazaretianemanuel@yahoo.com (E.N.); catalin.dumitras@tuiasi.ro (C.D.); 7 Physics Department, “Gheorghe Asachi” Technical University, Prof. Dr. Docent Dimitrie Mangeron Rd., No. 59A, 700050 Iasi, Romania; magop@tuiasi.ro (M.A.); cristina.rusu@tuiasi.ro (C.M.R.); 8 Academy of Romanian Scientists, 3 Ilfov, 050044 Bucharest, Romania

**Keywords:** mesoporous silica, modified drug delivery, metformin, MCM-48, diabetes mellitus, dissolution tests

## Abstract

**Background**: This study explored the potential of MCM-48 mesoporous silica matrices as a drug delivery system for metformin hydrochloride, aimed at improving the therapeutic management of type 2 diabetes mellitus. The objectives included the synthesis and characterization of MCM-48, assessment of its drug loading capacity, analysis of drug release profiles under simulated physiological conditions, and the development of a multifractal dynamics-based theoretical framework to model and interpret the release kinetics. **Methods**: MCM-48 was synthesized using a sol–gel method and characterized by SEM-EDX, TEM, and nitrogen adsorption techniques. Drug loading was performed via adsorption at pH 12 using metformin hydrochloride solutions of 1 mg/mL (P-1) and 3 mg/mL (P-2). In vitro dissolution studies were conducted to evaluate the release profiles in simulated gastric and intestinal fluids. A multifractal dynamics model was developed to interpret the release kinetics. **Results**: SEM-EDX confirmed the uniform distribution of silicon and oxygen, while TEM images revealed a highly ordered cubic mesoporous structure. Nitrogen adsorption analyses showed a high specific surface area of 1325.96 m²/g for unloaded MCM-48, which decreased with drug loading, confirming efficient incorporation of metformin hydrochloride. The loading capacities were 59.788 mg/g (P-1) and 160.978 mg/g (P-2), with efficiencies of 99.65% and 89.43%, respectively. In vitro dissolution studies showed a biphasic release profile: an initial rapid release in gastric conditions followed by sustained release in intestinal fluids, achieving cumulative releases of 92.63% (P-1) and 82.64% (P-2) after 14 hours. The multifractal dynamics-based theoretical release curves closely matched the experimental data. **Conclusions**: MCM-48 mesoporous silica effectively enhanced metformin delivery, offering a controlled release profile well-suited for type 2 diabetes management. The multifractal theoretical framework provided valuable insights into drug release dynamics, contributing to the advancement of innovative drug delivery systems.

## 1. Introduction

The development of advanced drug delivery systems (DDSs) has become one of the most important aspects of modern pharmacology and nanomedicine. These systems are designed to optimize the pharmacokinetics of drugs by controlling the release rate, enhancing the stability of the drug, and targeting specific tissues, thus improving therapeutic efficacy and reducing side effects [1]. Among the diverse array of DDS, mesoporous silica materials, especially those from the MCM-48 family, have gained considerable attention due to their well-defined pore structures, large surface area, and functionalization potential. These materials offer unique advantages in drug delivery, including their ability to load a high amount of active substance and to release it in a controlled manner, which is essential for chronic disease management, such as type 2 diabetes mellitus [2,3,4,5,6,7].

Metformin hydrochloride, the most widely prescribed oral antidiabetic agent, is the focus of this study. Despite its effectiveness in controlling blood glucose levels, its therapeutic application is often hindered by issues related to its solubility and bioavailability [8,9]. Thus, improving the drug delivery and release profiles of metformin can enhance its clinical outcomes. Mesoporous silica matrices, with their structural characteristics, have shown potential in overcoming these limitations by facilitating the controlled release of drugs in gastrointestinal fluids, which is crucial for maintaining stable blood glucose levels throughout the day [10].

MCM-48 is a well-known mesoporous material that exhibits a cubic pore structure, which offers significant advantages over other silica materials like MCM-41. The unique three-dimensional channel system of MCM-48 increases the surface area and pore volume, making it highly effective in loading and releasing various active compounds [11,12,13]. The high stability, chemical resistance, and tunable properties of MCM-48 make it an ideal candidate for use in drug delivery systems. Moreover, its ability to encapsulate metformin hydrochloride, a cationic drug, is particularly enhanced by the presence of silanol groups on the surface of the silica matrix, which interact favorably with the drug molecules [14,15,16].

The existing body of research on mesoporous silica drug delivery systems has shown that the drug release profiles can be controlled by manipulating various parameters such as pore size, surface functionalization, and the nature of the drug itself. However, the release dynamics are complex and depend on several factors, including the concentration of the drug, pH conditions, and the interaction between the drug and the matrix. While many studies have successfully demonstrated the potential of MCM-48 as a drug carrier, there is still a need to establish more accurate models to predict and control the release behavior of drugs from these systems [10].

A variety of semi-empirical models such as Higuchi, Korsmeyer-Peppas, and first-order kinetics have been employed to describe the release profiles of drugs from mesoporous silica matrices. However, these models often fail to fully explain the complexities of drug release mechanisms, especially in systems with intricate structural dynamics like mesoporous silica. To address these challenges, we propose the integration of multifractal theory into the drug release dynamics. The multifractal model offers a more refined approach by describing the drug release kinetics through continuous and non-differentiable curves, which may better reflect the real-world complexities of drug release from mesoporous matrices [17,18,19].

In this study, we aim to explore the use of MCM-48 mesoporous silica matrices for the controlled release of metformin hydrochloride. Our objectives include the synthesis and characterization of MCM-48, the loading of metformin, and the evaluation of the release profiles under simulated physiological conditions. We also employ a theoretical framework based on multifractal analysis to describe the drug release dynamics, offering a more robust method for modeling and predicting release patterns.

The main contributions of this research are twofold: (1) it provides a deeper understanding of the role of mesoporous silica in modifying the release profiles of metformin hydrochloride, and (2) it introduces a novel theoretical approach using multifractal dynamics to more accurately model the release behavior of drugs from such systems. We hypothesize that MCM-48, with its unique pore structure and high loading capacity, will provide an ideal matrix for controlling metformin release, improving its therapeutic performance. Furthermore, the integration of multifractal analysis will provide new insights into the complexity of drug release processes, contributing to the development of more efficient DDS.

The remainder of the article presents the synthesis and characterization of MCM-48, followed by the experimental setup for drug loading and release studies. We also discuss the theoretical model employed and compare the results with experimental data. Finally, we conclude with an analysis of the potential implications for improving the management of type 2 diabetes mellitus through enhanced drug delivery systems.

## 2. Material and Methods

### 2.1. Materials

A test experiment was performed using the following reagents: metformin hydrochloride (97% purity, C_4_H_11_N_5_·HCl; Mr = 165.6) purchased from Sigma Aldrich Chemie Gmbh (Steinheim, Germany), tetraethyl orthosilicate (TEOS 98%) supplied by Merck (Darmstadt, Germany), cationic surfactant structuring—Hexadecyltrimethyl-ammonium bromide (CTAB, 98%, C_19_H_42_BrN; Mr = 364.46) purchased from Sigma Aldrich Chemie GmbH (Steinheim, Switzerland), sodium acetate (CH_3_COONa ≥ 99.0%) obtained from Silal Trading SRL (Bucharest, Romania), potassium dihydrogen phosphate (KH_2_PO_4_ ≥ 99.5%) obtained from Utchim SRL (Ramnicu Valcea, Romania), potassium chloride (KCl ≥ 99.0%) obtained from Chemical Company S.A. (Iasi, Romania), glacial acetic acid (CH_3_COOH, 99.9%) obtained from Chimreactiv SRL (Bucharest, Romania), hydrochloric acid (HCl ≥ 37.0%) and sodium hydroxide (NaOH, 98.5%) purchased from Chemical Company S.A. (Iasi, Romania), methanol (CH_3_OH, Mr = 32.04) for HPLC, ≥ 99.9% (Chromasolv™) purchased from Honeywell Riedel-de Haën (Seelze, Germany). Deionized water was prepared with an ELGA Purelab Ultra water system. The distilled water was obtained in the private laboratories with a GFL type 2004, no. 11918315J distiller (Burgwedel, Germany). All reagents were used without further purification.

### 2.2. Methods

#### 2.2.1. MCM-48 Mesoporous Silica Synthesis

For the synthesis of MCM-48, 5.2 g of CTAB was weighed into a watch glass, and then transferred to a 500 mL Berzelius beaker and dissolved in 240 mL deionized water and 100 mL ethyl alcohol. The mixture was magnetically stirred for 10 min until a clear, colorless solution was obtained. Subsequently, 24 mL of 25% NH_4_OH was added, followed by homogenization for 2 min. Afterward, 7.2 mL of TEOS was gradually introduced dropwise over a period of 5 to 7 min. Ultrasonication was initiated at the time of TEOS addition, set at 25% amplitude with a 3 s on/1 s off cycle, and continued for 1 h (80 min). Gentle agitation was performed at 15 min intervals throughout the process.

After completion of the ultrasonication program and obtaining the silicate matrix, the temperature of the reaction mixture was determined. Separation of MCM-48 from the supernatant was carried out by centrifugation at 4000 rpm for 10 min. After each centrifugation step, the supernatant was gently separated (by decantation). The final step involved washing the MCM-48 using deionized water, ethyl alcohol, and a 1:1 water–alcohol mixture, performing 3 to 5 washing cycles. The washed MCM-48 was then dried in an oven at 60 °C for 48 to 72 h. Following the drying process, the material undergoes calcination at 550 °C for 8 h to eliminate organic compounds and to establish the mesoporous structure.

#### 2.2.2. Metformin Immobilization

The loading of the mesoporous silicate matrix with metformin hydrochloride was carried out by adsorption. Metformin hydrochloride was dissolved in aqueous solutions at predefined pH = 12, at a concentration of 1 and 3 mg/mL. Silica matrix (0.5 g) was weighed on an analytical balance, and was then oven-dried at 200 °C for 2 h to remove residual moisture and subsequently cooled in a controlled atmosphere in an exicator. The dried matrix was in contact with 60 mL aqueous solution of metformin hydrochloride under magnetic stirring for 24 h. After 24 h, the suspensions were centrifuged at 4000 rpm for 7 min to separate the loaded matrix from the supernatant, and the loaded matrix was dried at room temperature or in an oven at controlled temperature (25–30 °C).

#### 2.2.3. Quantitative Determination of Metformin

The determination of the loading degree, defined as the ratio of the active substance to one gram of the silica matrix, was performed by calculating the difference between the initial amount of metformin hydrochloride in the loading solution and the amount of metformin hydrochloride quantitatively measured in the filtered supernatant after contact with the matrix. For the quantitative determinations, samples were injected to HPLC using the method developed, optimized, and validated. The optimized working conditions of the method consist in the use of a mobile phase composed of 0.02 M acetate buffer (pH = 3)/methanol (70/30, *v*/*v*). The working temperature was 35 °C. The column used was Thermoscientific ODS Hypersyl ™ (Vilnius, Lithuania) 250 mm × 4.6 mm, 5 µm; the injection volume was 20 µL and chromatogram detection was set at a wavelength of 235 nm [9].

#### 2.2.4. Scanning Electron Microscopy (SEM) Coupled with Energy Dispersive X-Ray Spectroscopy (EDX-SEM)

In this microstructural analysis technique using scanning electron microscopy (SEM) on solid materials like mesoporous silicate matrices, an electron beam was scanned across the outer surface of the matrix. SEM-EDX measurements were conducted using a Quanta 200 scanning electron microscope (FEI Company, Hillsboro, OR, USA). Both metformin hydrochloride-loaded and unloaded silicate matrices were analyzed to obtain information about their degree of loading.

#### 2.2.5. Transmission Electron Microscopy (TEM) Analysis

Transmission Electron Microscopy (TEM) investigations were performed using a HT7700 HITACHI microscope (Hitachi High-Technologies Corporation, Tokyo, Japan) operated in high-contrast mode at 100 kV accelerating voltage. The samples were prepared by direct depositing solid particles of silicate matrices on a 300-mesh carbon-coated copper grid Ted Pella (Redding, CA, USA).

#### 2.2.6. Nitrogen Sorption Isotherms

Texture and porosity properties were analyzed by the nitrogen adsorption–desorption method. The determination of the specific surface area by the BET method was based on the measurement of the amount of nitrogen adsorbed or desorbed on the surface of solids, porous or non-porous. The unloaded sample (MCM-48) and the loaded samples (P-1 and P-2) were analyzed on the Quantachrome Nova 2200 Instrument and Pore Size Surface Area Analyzer (Quantachrome Instruments, Odelzhausen, Germany) by recording the sorption isotherms. The graphical representation of the amount of gas adsorbed by the sample as a function of gas pressure under constant temperature conditions is called the adsorption isotherm. In the next step, the obtained information and data were processed according to adsorption theories, and the BET specific surface area in m^2^/g (calculated by Brunauer–Emmett–Teller method) was calculated. The total pore volume was determined by measuring the volume of adsorbed liquid nitrogen [20].

#### 2.2.7. In Vitro Dissolution Tests

The dissolution test was performed on the SR 8 Plus Series (AB & L Jasco, Cluj-Napoca, Romania), type 2 (paddle), following the protocol: simulated gastric fluid dissolution medium (pH = 1.2)—50 mL for the first 2 h, replaced subsequently with simulated intestinal fluid medium (pH = 6.8) for 12 h. The temperature was kept constant at 37 ± 0.5 °C, stirring was 50 rpm. Samples were collected at the following sampling times: 0.5, 1, 2, 3, 4, 5, 6, 7, 8, 9, 10, 11, 12, 13, and 14 h. At each sampling time, an aliquot of 1 mL was replaced with the same volume of fresh medium heated to 37 °C. The collected samples were filtered and injected to HPLC by using a laboratory validated analytical method to quantitatively determine metformin hydrochloride [10].

#### 2.2.8. Analysis of In Vitro Drug Release Kinetics

Analysis of the release kinetics of metformin hydrochloride from MCM-48 was performed by fitting on four mathematical models (zero-order and first-order kinetics, Higuchi and Korsmeyer-Peppas models), following data obtained from the in vitro dissolution study. Data were presented as mean ± standard deviation and were considered statistically significant at *p* < 0.05. A reliable prediction model has an R^2^ value close to 1.

The following mathematical equations were used:

Zero-order kinetic:

(1) $$M_{t}=K_{0}\cdot t$$

First-order kinetic:

(2) $$M_{t}=100\cdot \left(1-e^{-kt}\right)$$

Higuchi model:

(3) $$M_{t}=K_{H}\cdot t^{0.5}$$

Korsmeyer-Peppas model:

(4) $$M_{t}=K_{p}\cdot t^{n}$$

where $M_{t}$ is the amount of drug substance released at time *t*, $K_{0}$ is the zero-order rate constant of the drug release rate, *k* is the first-order yield rate constant, $K_{H}$ is the yield rate constant of the Higuchi model (Higuchi release constant), $K_{p}$ is the Korsmeyer–Peppas release rate constant, *n* is an exponential factor (an indicator of the drug release mechanism), and *t* is the time.

## 3. Results

The mesoporous MCM-48 matrix was synthesized via a chemical process utilizing an ultrasonic method, followed by the adsorption-based incorporation of metformin from aqueous solutions at varying concentrations and a controlled pH. High-performance liquid chromatography (HPLC) analysis quantified the metformin loading as 59.788 mg/g of silica (P-1) and 160.978 mg/g of silica for sample P-2, respectively, achieving optimal loading efficiencies exceeding 89% (Table 1).

**Figure 1:**
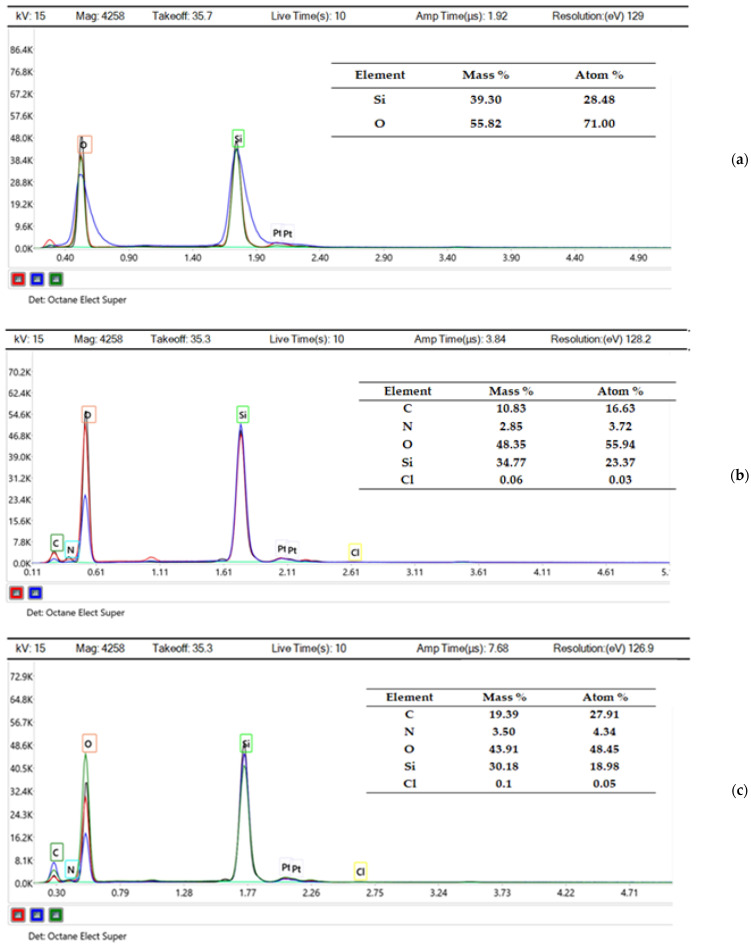
Elemental composition (EDX spectra) of MCM-48 sample unloaded (**a**); MCM-48 sample P-1 (**b**); and MCM-48 sample P-2 (**c**).

SEM-EDX analysis demonstrated that the pure MCM-48 matrices contained an average silicon (Si) and oxygen (O) content of 39.30% and 55.82% by weight, respectively, corresponding to 28.48% and 71.00% in atomic percentage (Figure 1).

The loading of MCM-48 with metformin hydrochloride in sample P-1 (1 mg/mL, pH = 12) indicates a significant increase in carbon (C) and nitrogen (N) content, which reflects the optimal loading efficiency (Figure 1). For sample P-2 (3 mg/mL, pH = 12), a proportional increase in C and N content was observed, correlated with the degree of loading, resulting from effective dispersion of the active substance in the mesoporous matrix (Figure 1).

**Figure 2:**
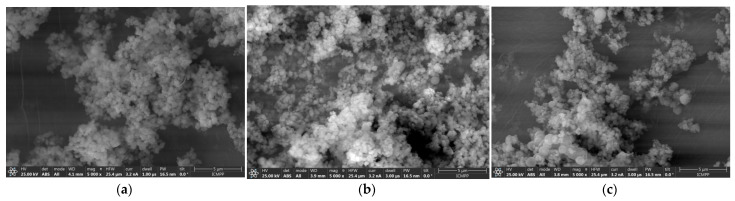
SEM images: MCM-48 sample unloaded (**a**); MCM-48 sample P-1 (**b**); and MCM-48 sample P-2 (**c**).

SEM images revealed quasi-spherical particles of different sizes for the unloaded matrices, with a higher tendency to conglomerate and a small particle size. The conglomeration of the mesoporous MCM-48 matrices may influence the loading capacity, distribution, and interaction with metformin molecules. In the case of the metformin hydrochloride-loaded matrices (P-1 and P-2), quasi-spherical particles were observed sticking to each other, resembling a grape cluster, indicating a high degree of aggregation (Figure 2).

**Figure 3:**
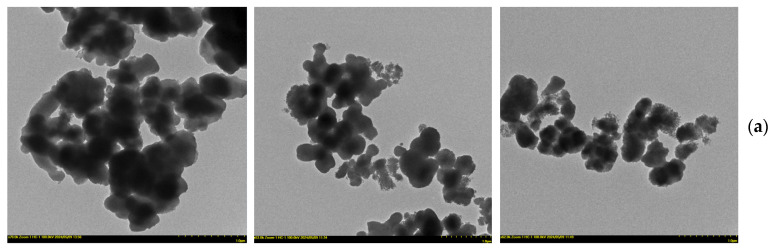

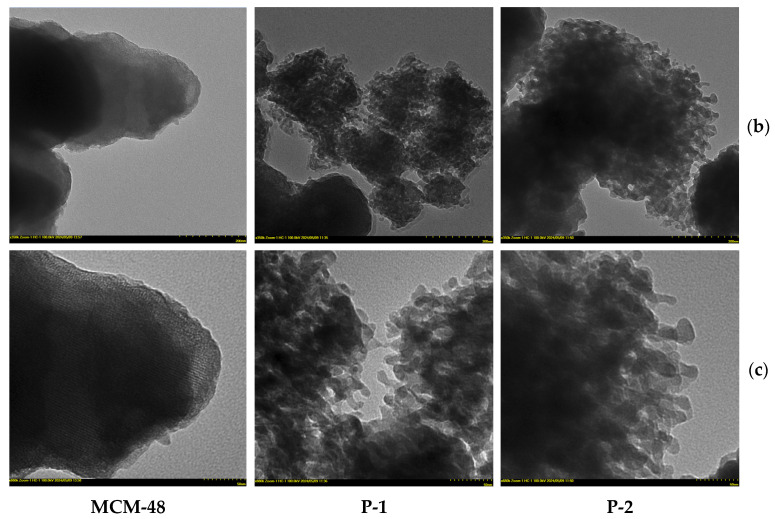
TEM images of MCM-48 sample unloaded; MCM-48 sample P-1 and MCM-48 sample P-2: (**a**) 1 µm, (**b**) 200 nm, and (**c**) 50 nm.

In order to extend the information about the structure of the silicate matrices, transmission electron microscopy (TEM) images were added to the data. An overview of the MCM-48 particles is shown in Figure 3a (1 μm). One can notice clusters of dispersed quasi-spherical particles of relatively uniform size. Figure 3b (200 nm) reveals greater clarity of the quasi-spherical shape for the MCM-48 particles. At this magnification, it can be seen that the particles tend to form clusters, and the sizes of the individual particles appear to be fairly uniform, around 100–200 nm in diameter. Figure 3c (50 nm) is a detailed image showing the surface structure of the MCM-48 particles. The surface appears to have a uniform texture, indicating a porous structure characteristic of this type of mesoporous material.

In the first step, nitrogen adsorption isotherms were performed, which provide essential information about the structural and textural characteristics of porous materials, such as specific surface area, pore volume, pore size distribution, and porosity type. The specific pore surface area provides information about the surface area available for the adsorption of active molecules. If for the uncharged matrix MCM-48 the specific surface area is 1325.96 m^2^/g, for the loaded samples (from aqueous solutions at pH = 12, concentration of 1 and 3 mg/mL), i.e., P-1 and P-2, we notice a decrease in the specific surface area, correlated with the degree of loading of the samples, proportional to the loading efficiency, so that for P-1 we have 54.005 m^2^/g (efficiency of 99.65%), and for P-2 we find a specific surface area of 137.19 m^2^/g (89.43%) (Table 1 and Table 2). We find that the loading yield decreases by 10.22% between the concentration of 1 mg/mL versus 3 mg/mL, but the loading degree of sample P-2 (160.978 mg/g silica) is 269% higher compared to sample P-1 (59.788 mg/g silica) (Table 1).

The correlation coefficient indicates the similarity with the BET experimental model, being all the better as the value approaches 1, and from the experimental data in Table 2, it can be seen that the value (r^2^) is at least 0.9981.

The external surface area excludes micropores and generally a decrease in the external surface area of the matrices is observed upon loading compared to unloaded matrices.

**Figure 4:**
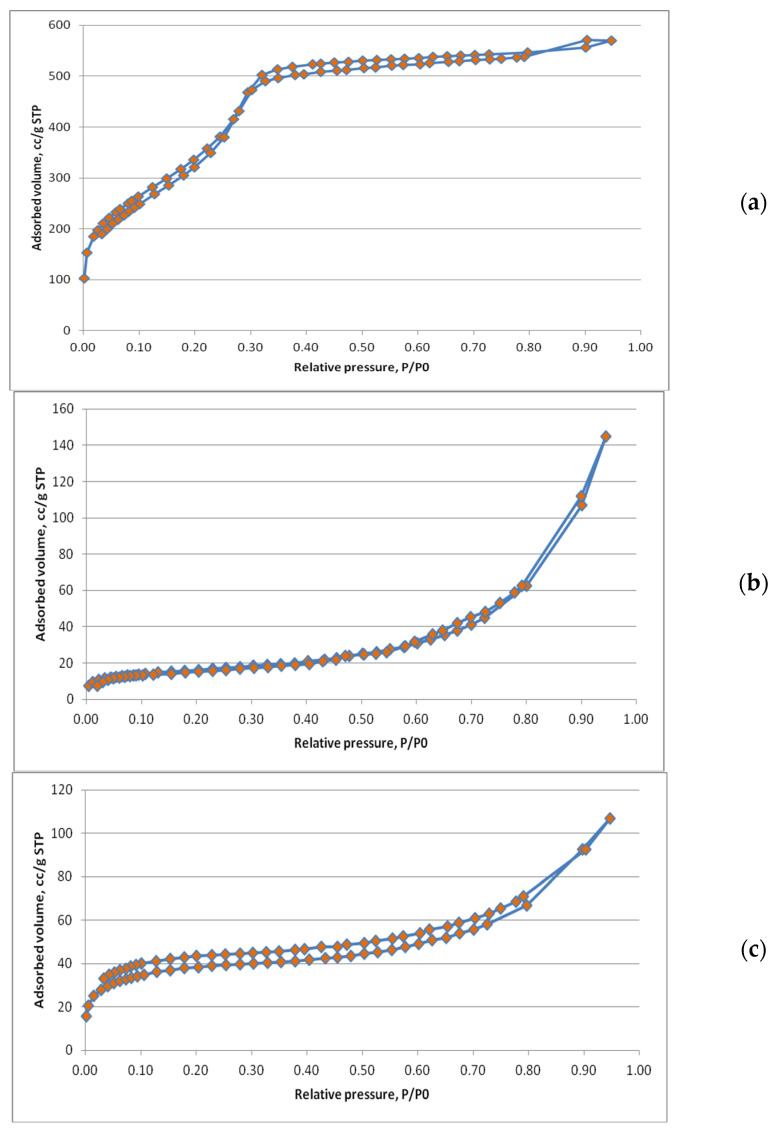
Nitrogen adsorption isotherm of MCM-48 sample unloaded (**a**); MCM-48 sample P-1 (**b**); and MCM-48 sample P-2 (**c**).

The overall loading capacity was assessed by the total pore volume (micro-, meso-, and macro-pores), which generally decreases upon loading. Thus, sample P-2, which had the highest loading (160.978 mg/g silica), was also characterized by the lowest total pore volume value (0.166 cm^3^/g). The nitrogen adsorption isotherms are presented in Figure 4.

**Figure 5:**
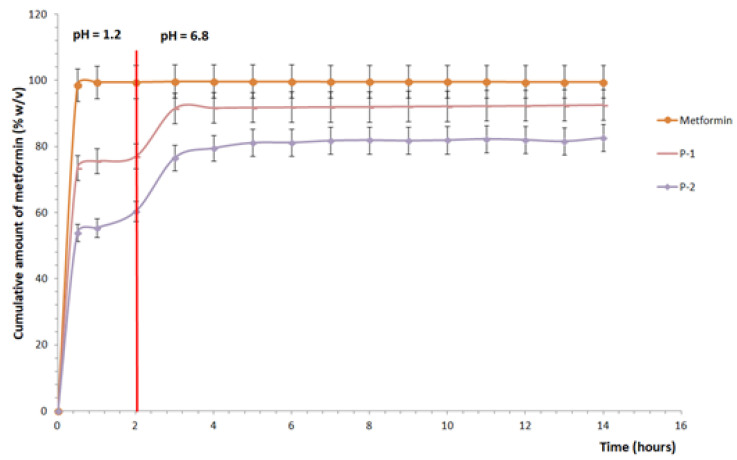
In vitro dissolution release of metformin from mesoporous silica.

In the in vitro dissolution test study for MCM-48 samples loaded with metformin hydrochloride at pH = 12 at two concentrations (1 and 3 mg/mL), the experimental data are shown in Figure 5.

In simulated gastric medium, at pH = 1.2, the percentage of metformin release for the pharmaceutical form released after 30 min, at the first sampling is 73.54% (for sample P-1) and 53.92% (for sample P-2), with a maximum concentration of 77.08% (P-1) and 60.42% (P-2), obtained after 2 h. At the end of the 2 h, the simulated gastric dissolution medium was changed to simulate intestinal fluids (pH = 6.8) where at the first sampling, after 3 h, there was an increase in the dissolving capacity of metformin hydrochloride observed in both samples. At the end of the assay, after 12 h in simulated intestinal medium, the percentage amount of metformin hydrochloride yielded was between 92.63% (P-1) and 82.64% (P-2).

In the case of the free metformin hydrochloride, used as a control, with conventional release, a total yield of 98.68% was observed after 30 min, and at the end of the test the recovery was 99.62%.

The criterion for the selection of the model indicating the metformin hydrochloride yield kinetics was the correlation coefficient R^2^ (Table 3).

The Korsmeyer–Peppas model best described the kinetics of metformin hydrochloride release from the mesoporous MCM-48 matrices, regardless of the variation in concentrations (1 and 3 mg/mL), with a correlation coefficient R^2^ greater than 0.8 in both cases. The n and K_P_ values are listed in Table 3. The samples taken in the work showed an n parameter value less than 0.43, indicating a Fickian diffusion.

### Theoretical Design

If any drug delivery system can be assimilated—both structurally and functionally—to mathematical objects of fractal/multifractal type [11,12], then the release kinetics can be described based on the Multifractal Theory of Motion [11,12], through continuous and non-differentiable curves (fractal/multifractal curves). From such a perspective, in accordance with Appendix A, the fractal/multifractal differential equation of the drug release rate becomes functional, in the following form:

(5) $$\frac{\partial \Phi }{\partial t}+V\frac{\partial \Phi }{\partial z}-D\frac{\partial^{2}\Phi }{\partial z^{2}}=0$$

The meanings of the quantities from Equation (5), types of drug release dynamics which can be induced by means of fractalization/multifractalization associated with Markovian and non-Markovian type stochastic processes, are largely discussed in Appendix A in the context of the Multifractal Theory of Motion [11,12].

In such a context, some implications become obvious.

(a) If, in Equation (5), we operate with the fractal/multifractal transformation:

(6) $$\Phi \left(z,t\right)=\theta \left(z,t\right)exp\left(\frac{Vz}{2D}-\frac{V^{2}t}{4D}\right)$$

it becomes

(7) $$\frac{\partial \theta }{\partial t}=D\frac{\partial^{2}\theta }{\partial z^{2}}$$

The differential Equation (7) is reduced to the standard one for the case of drug release dynamics on monofractal manifolds, dynamics which are described through fractal curves with the fractal dimension $D_{F}=2$ (Peano-type curves [11,12]).

(b) Assuming that the plane z=0 constitutes the basis of a semi-finite homogenous layer, which was initially at the rate $\theta_{0}$ for z>0, then the solution for the distribution of the drug release along z in the time t, in accordance with Appendix B, will be in the following form:

(8) $$\Phi \left(z,t\right)=\frac{\theta_{0}}{2}\exp\left(-\frac{V^{2}t}{D}\right)+\frac{\theta_{0}}{2}\exp\left(-\frac{V^{2}t}{D}\right)\;\;\;\;\;\;\;\;\;\;\;\;\;\cdot \left\{erf\left[\frac{\left(z-Vt\right)}{{\left(4Dt\right)}^{\frac{1}{2}}}\right]+erf\left[\frac{\left(z+Vt\right)}{{\left(4Dt\right)}^{\frac{1}{2}}}\right]\right\}$$

where erf(x) is the Laplace function imparted as follows:

(9) $$\mathrm{erf}\left(x\right)=\frac{1}{2\sqrt{\pi }}\int_{0}^{\infty }\exp\left(-x^{2}\right)dx$$

values of this function being tabulated.

**Figure 6:**
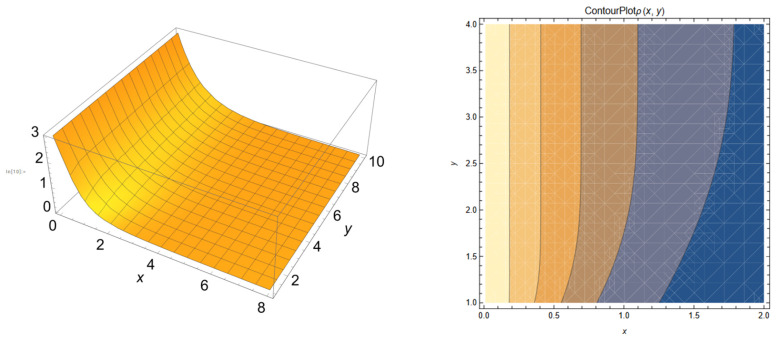

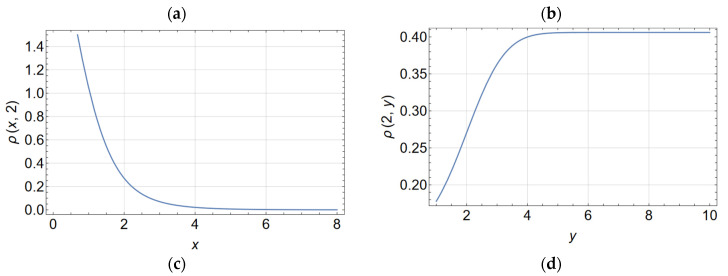
Release rate dependences: (**a**) 3D plot in non-dimensional coordinates; (**b**) 2D plot in non-dimensional coordinates; (**c**) $\phi \equiv \rho \left(x,2\right)$; (**d**) $\phi \equiv \rho \left(2,y\right)$.

In Figure 6a–d, we present the dependences of the release rate.

**Figure 7:**
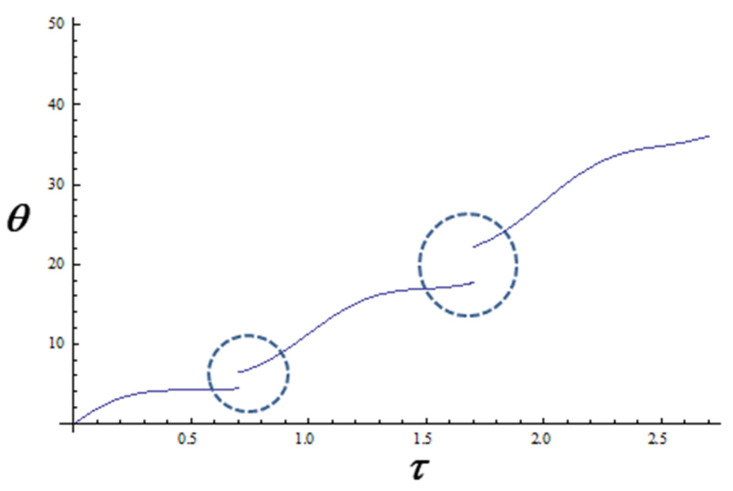
Drug release kinetics for various fractality degrees expressed as different resolution scales: 1, 1.5, and 2 (in coordinates ϕ≡θ, y≡τ). The dot circle indicates where the resolution scale changes.

Since *y* is related to the temporal component, it results that our theoretical curves for a set fractality degree (see Figure 7) are in good accordance with the experimental drug release curves.

Moreover, if the fractality degree is changed, a new curve (with the same format) can be obtained, at another scale (similar to the curves from Figure 5).

## 4. Discussion

The synthesis of MCM-48 was carried out using tetraethyl orthosilicate (TEOS) as the precursor for matrix synthesis. TEOS, by hydrolysis, becomes hydrophilic and is released as a monomer or oligomer into the aqueous medium and is adsorbed by the surfactant (CTAB) through electrostatic attraction. Hydrolysis and condensation occur simultaneously, forming a silica shell around the micelles. Once the surfactant is removed (by solvent extraction or by calcination), various geometries (usually hexagonal, cubic, or lamellar) are formed, usually constituting separate channels or cavities and supported by walls of amorphous silica [21]. MCM-48 has a three-dimensional cubic, bi-continuous structure with pore sizes between 1.6 and 3.8 nm [22].

Metformin hydrochloride, a cationic compound containing two guanidine groups, has two pKa values (3.1 and 13.8) so it exists in neutral, mono-, or di-protonated form depending on the pH of the environment; therefore, it can exist in a di-protonated form at pH < 3.1, mono-protonated at pH 3.1–13.8, and neutral at pH > 13.8 [23,24]. At pH = 12, the solution is strongly alkaline and deprotonation of the silanol groups simultaneously occurs, which results in the formation of negative charges on the surface of the mesoporous material. Thus, the -O- groups on the silica surface can interact favorably with the protonated amine cationic groups of metformin, exhibiting a higher affinity for adsorption on mesoporous materials. The preferential loading in MCM-48 is explained by the cubic three-dimensional structure with a network of interconnected pores, which may facilitate a larger and more uniform contact with metformin molecules. MCM-48 has smaller pore sizes and a denser network of channels, which may lead to a higher loading capacity and a more uniform distribution of the active molecule [11].

SEM-EDX compositional analysis of the samples for unloaded matrices (MCM-48) and the loaded matrices (P-1 and P-2) yields data on the concentrations of the relevant chemical elements. Oxygen dominates the atomic composition due to its abundance in the silica structure. Relatively higher oxygen and lower silica content are found for MCM-48, which may indicate a possible higher presence of -OH groups on the surface, which also explains the increased ability of the matrix to bind metformin hydrochloride molecules. Metformin contains amino functional groups that can form hydrogen bonds with the -OH groups on the surface of the mesoporous silica. After loading, there is a significant increase in carbon and nitrogen as well as the secondary chlorine content, which indicates the presence of organics and efficient loading with metformin hydrochloride.

SEM analysis provides high-resolution images for texture, morphology, and matrix structure. The SEM images revealed quasi-spherical particles that provide a high specific surface area, favoring efficient interaction with metformin hydrochloride, but also a higher probability of agglomeration. Also, during the loading process, the metformin solution can induce capillary condensation in the pores of the matrix, leading to particle agglomeration. After loading, the drying process may favor the attraction of quasi-spherical particles towards each other, thus forming agglomerates [25,26].

The observed agglomerations clustered in clusters of varying sizes may reflect the efficiency and distribution mode of metformin in the mesoporous matrix. Thus, the particle aggregation may indicate an uneven distribution of metformin, where certain areas of the matrix have a higher loading density compared to others. This may be influenced by the initial concentration of metformin solution and loading conditions. In addition, agglomerated particles can reduce solvent accessibility to internal pores, affecting the controlled release of metformin in pharmaceutical applications [26,27].

In general, as a delivery system for active molecules, mesoporous silica materials exhibit a high dependence on the nature of the pores in terms of the shape, size, and connectivity of the mesoporous structure and its geometry. Differences were noticed in samples obtained by preparation from aqueous solutions at pH = 12. In this case, as the metformin molecule was not protonated, it interacted similarly with both matrices, so that the release profiles were also similar, reaching after 14 h maximum percentages ranging from 82.64 to 92.63%.

It can be observed that after 2 h, more than half of the total maximum percentage of metformin was released into the gastric medium. This is justified by the first-step release of metformin bound superficially to the matrix surface or external canaliculi, and subsequently, the active substance was slowly released from the canaliculi and internal pores of the matrix. Thus, it was observed that during the next 12 h in the intestinal environment, the release occurred slowly (an increase of 15–25%). For sample P-1, it went from 77.08% maximum percentage released in the gastric environment to 92.63% maximum percentage released in the intestinal environment. The experimental data suggest that formulations that release metformin in multiple steps, both in the stomach and in the intestine, could optimize both immediate and long-term absorption, thereby improving therapeutic efficacy.

On the other hand, by assimilating this type of drug delivery system, both structurally and functionally, with a multifractal, through the variation of the fractal degree, we can obtain, in non-dimensional coordinates, drug release curves that accurately fit our experimental data. More precisely, the fractal degree (determined both by the scale resolution and the fractal dimension of the drug release curves) must be identified with the drug release rate.

## 5. Conclusions

This study aimed to experimentally analyze the influence of the concentration of metformin hydrochloride solution at pH = 12 in the loading process of mesoporous matrices (MCM-48). A mesoporous silica matrix (MCM-48) was synthesized and physicochemically characterized. Metformin was then immobilized via adsorption from an aqueous solution at pH = 12 using magnetic stirring for 24 h, resulting in a loading degree of 59.788 mg/mL for sample P-1 and 160.978 mg/mL for sample P-2. Increasing the concentration of metformin hydrochloride from 1 mg/mL to 3 mg/mL resulted in a significant increase in the loading capacity of MCM-48, with an approximate enhancement of 270%. This highlights the significant impact of concentration on the immobilization process. The pore structure of the silica matrix plays a crucial role in the adsorption capacity and release characteristics of metformin. The development of mesoporous silica nanoparticles loaded with metformin holds promise for improving drug delivery systems, optimizing biopharmaceutical properties, reducing side effects, and enhancing therapeutic compliance in the management of type 2 diabetes mellitus.

From a theoretical perspective, if we assimilate this specific drug delivery system, both structurally and functionally, with a multifractal, then its dynamics can be described through continuous and non-differentiable curves. In such a context, we have obtained theoretical drug release curves that are in good accord with the experimental ones.

## Figures and Tables

**Table 1:** Experimental data on the degree of loading of mesoporous silicate matrices.

Sample Code	Concentration Sol. (mg/mL)	pH	Metformin Load Grade (mg/g)	Metformin Loading Yields (wt%)
P-1	1	12	59.788	99.65
P-2	3	12	160.978	89.43

**Table 2:** Textural properties of the prepared mesoporous silicate matrices.

Sample Code	Specific Surface Area S_BET_ (m^2^/g)	Correlation Coefficient with BET Model (r^2^)	External Surface Area (m^2^/g)	Total Pore Volume (cc/g)
P-1	54.005	0.9998	51.084	0.224
P-2	137.19	0.9997	115.661	0.166
MCM-48	1325.96	0.9981	1325.96	0.881

S_BET_: Brunauer–Emmett–Teller surface area analysis.

**Table 3:** Parameter values of the kinetic release.

Kinetic Model	Model Coefficients	Mesoporous Silica	
		P-1	P-2
Zero-order	K_0_	2.7983	3.0966
	R^2^	0.3102	0.4409
First-order	K_1_	0.1156	0.0852
	R^2^	0.5231	0.5958
Higuchi	K_H_	15.271	15.9950
	R^2^	0.5355	0.6810
Korsmeyer–Peppas	n	0.0760	0.1433
	K_P_	78.1808	59.7585
	R^2^	0.8028	0.8547

## Data Availability

All the data are presented in the manuscript.

## Appendix A

If the polymer–drug system can be assimilated both structurally and functionally to mathematical objects of fractal/multifractal type [19,28,29,30], any of its dynamics can be described through the Multifractal Theory of Motion [18,19] by means of continuous and non-differentiable curves (fractal/multifractal curves). Then, the scale covariant derivative [14,15] becomes functional

(A1) $$\frac{\hat{d}}{dt}=\partial t+{\hat{V}}^{e}\partial_{e}+D^{ie}\partial_{i}\partial_{e},\;\;i,e=1,2,3$$

where the subsequent notations are employed:

(A2) $$\partial_{t}=\frac{\partial }{\partial t},\;\;\partial_{e}=\frac{\partial }{\partial x^{e}},\;\;\partial_{i}\partial_{e}=\frac{\partial }{\partial x^{i}}\frac{\partial }{\partial x^{e}}\;\;{\hat{V}}^{e}=V_{D}^{e}-iV_{F}^{e},\;\;i=\sqrt{-1}\;D^{ie}={\frac{1}{4}\left(dt\right)}^{\left[\frac{2}{f\left(\alpha \right)}\right]-1}\left(d^{ie}+i{\bar{d}}^{ie}\right)\;d^{ie}=\left(\lambda_{+}^{i}\lambda_{+}^{e}-\lambda_{-}^{i}\lambda_{-}^{e}\right),\;\;{\bar{d}}^{ie}=(\lambda_{+}^{i}\lambda_{+}^{e}+\lambda_{-}^{i}\lambda_{-}^{e})$$

In Equations (A1) and (A2), $x^{e}$ defines the spatial coordinates, depicted by continuous and nondifferentiable mathematical functions which rely on the scale resolution. The temporal coordinate, *t*, is depicted by continuous and differentiable mathematical functions which do not rely on scale resolution. $V_{D}^{e}$ defines the differentiable velocity (i.e., the velocity at a differential scale resolution), while $V_{F}^{e}$ illustrates the nondifferentiable velocity (i.e., the velocity at nondifferentiable scale resolution). $\lambda_{+}^{e}$ and $\lambda_{-}^{e}$ are particular constants of the differentiable–nondifferentiable scale transition coupled with the forward and backward SMA dynamics, respectively. The singularity spectrum of order α, identified through $f\left(\alpha \right)$, is defined by $\alpha =\alpha \left(D_{F}\right)$, where $D_{F}$ represents the fractal dimension of the motion curves of the structural units of any polymer–drug system [31,32,33].

In this frame of reference, employing the singularity spectrum of order α to characterize drug release kinetics has obvious advantages:

1. The dynamics pertaining to the structural units of the polymer–drug system may reveal a dominant fractal dimension, which may help in pinpointing a global release pattern which is consistent with a particular global polymer–drug system structure and functionality.
2. The dynamics pertaining to the structural units of the polymer–drug system could include a “collection” of fractal dimensions, which may help in pinpointing local release patterns that are compatible with particular, zone-specific polymer–drug structures and functions.
3. By means of the α-order singularity spectrum, one can pinpoint universality classes in the domain of drug release dynamics, even when the attractors related to said dynamics have distinct aspects.

The explicit configuration of the tensor $D^{ie}$, i.e., the one connected to the fractal-non-fractal/multifractal-non-multifractal scale transitions, is prescribed by fractalization through stochasticity. A very usual procedure of fractalization/multifractalization is by means of stochastic processes [11,12,22]. In such a conjecture, it is essential to explain that the stochastic or chaotic processes employed in the study of drug release dynamics can be split into two categories. The first category comprises homogeneous drug release dynamics, which are described through a single global fractal dimension and display the identical scaling properties in any time interval (known as fractal processes, such as fractional Brownian type processes). The second category entails drug release dynamics that can be depicted by employing multiple fractal dimensions and quantify strictly local singularities, known as multifractal processes.

As such, it is possible to identify the subsequent types of drug release dynamics:

- Multifractal drug release dynamics by means of Markov stochasticity, dictated through the subsequent boundaries [11,12]:
  (A3) $$\lambda_{+}^{i}\lambda_{+}^{e}=\lambda_{-}^{i}\lambda_{-}^{e}=-2\lambda \delta^{ie}$$
  where λ is a diffusion-type coefficient coupled with the multifractal–non-multifractal scale transition and $\delta^{ie}$ is the Kronecker pseudotensor. Therefore,
  (A4) $$D^{ie}\to i\lambda {\left(dt\right)}^{\left[\frac{2}{f\left(\alpha \right)}\right]-1}$$
  in a way that Equation (A1) emerges as below:
  (A5) $$\frac{\hat{d}}{dt}=\partial_{t}+{\hat{V}}^{e}\partial_{e}-i\lambda {\left(dt\right)}^{\left[\frac{2}{f\left(\alpha \right)}\right]-1}\partial^{e}\partial_{e}$$
- Multifractal drug release dynamics through non-Markov stochasticity dictated by the subsequent boundaries [3,4,5,6]:
  (A6) $$d^{ie}=4\alpha \delta^{ie},\;\;{\bar{d}}^{ie}=-4{\beta \delta }^{ie}\;$$
  where α and β are diffusion-type coefficients coupled with the multifractal-non-multifractal scale transition. As such, the following case results:
  (A7) $$D^{ie}=\left(\alpha -i\beta \right){\left(dt\right)}^{\left[\frac{2}{f\left(\alpha \right)}\right]-1},$$
  in a way that Equation (A1) emerges, as such:
  (A8) $$\frac{\hat{d}}{dt}=\partial_{t}+{\hat{V}}^{e}\partial_{e}-(\alpha -i\beta )\;{\left(dt\right)}^{\left[\frac{2}{f\left(\alpha \right)}\right]-1}\partial^{e}\partial_{e}$$

Now, presuming the functionality of the principle of scale covariance, based on which the laws of polymer–drug physics stay invariant regarding both spatial and temporal transformations and scale transformations, various conservation laws can be built.

For instance, if one utilizes the complex operator Equations (A8) upon the drug release rate Φ, then the conservation law has the following shape:

(A9) $$\frac{\hat{d\Phi }}{dt}=\partial_{t}\Phi +{\hat{V}}^{e}\partial_{e}\Phi -\left(\alpha -i\beta \right){\left(dt\right)}^{\left[\frac{2}{f\left(\alpha \right)}\right]-1}\partial^{e}\partial_{e}\Phi =0$$

or, described on resolution scales as such:

(A10) $$\partial_{t}\Phi +V_{D}^{e}\partial_{e}\Phi +\alpha \;{\left(dt\right)}^{\left[\frac{2}{f\left(\alpha \right)}\right]-1}\partial^{e}\partial_{e}\Phi =0$$

at differential scale resolutions, i.e., as below:

(A11) $$-V_{F}^{e}\partial \Phi -\beta \;{\left(dt\right)}^{\left[\frac{2}{f\left(\alpha \right)}\right]-1}\partial^{e}\partial_{e}\Phi =0$$

at non-differential scale resolutions. In the following, if the below notation is used:

(A12) $$V^{e}=V_{D}^{e}-V_{F}^{e}$$

the velocity related to the multifractal-non-multifractal scale transition, by summing up relations Equations (A10) and (A11), and the conservation law of the thermal field affiliated with the multifractal-non-multifractal scale transition is extracted as below:

(A13) $$\partial_{t}\Phi +V^{e}\partial_{e}\Phi +(\alpha -\beta )\;{\left(dt\right)}^{\left[\frac{2}{f\left(\alpha \right)}\right]-1}\partial^{e}\partial_{e}\Phi =0$$

In what follows, the consequences of this type of differential equation in modeling drug release dynamics are presented. For this reason, let us consider the differential Equation A(13) in the one-dimensional case and subjected to the conditions below:

(A14) $$V^{e}\equiv (0,0,V=const)$$

This then becomes as follows:

(A15) $$\frac{\partial \Phi }{\partial t}+V\frac{\partial \Phi }{\partial z}-D\frac{\partial^{2}\Phi }{\partial z^{2}}=0$$

where the notation is made:

(A16) $$D=(\beta -\alpha )\;{\left(dt\right)}^{\left[\frac{2}{f\left(\alpha \right)}\right]-1}$$

where *D* depends on the geometrical, physical, and scale resolution properties of the polymer–drug system.

## Appendix B

Let us consider the multifractal differential equation of drug release kinetics, in the following form:

(A17) $$\frac{\partial \theta }{\partial t}=D\frac{\partial^{2}\theta }{\partial z^{2}}$$

Based on the initial conditions and the boundary one:

(A18) $$\Phi \left(z,0\right)=\Phi_{0}=const\;\Phi \left(0,t\right)=0$$

the boundary conditions associated with θ are extracted as below:

(A19) $$\theta \left(z,0\right)=\theta_{0}\exp(\frac{Vz}{2D})\;\theta \left(0,t\right)=0$$

This issue can be restricted to one devoid of boundary conditions by expanding the solution to take into account the region z<0, where θ presumes an original distribution which mimics the boundary condition for z=0 at any time t>0. Simultaneously, it is possible to select the following:

(A20) $$\theta \left(z,0\right)=\theta (-z,0)$$

thus, for any expanded domain, the initial condition is stated as follows:

(A21) $$\theta \left(z,0\right)=\left\{\begin{array}{c} \theta_{0}\exp\left(\frac{Vz}{2D}\right),\;z>0\\ -\theta_{0}\exp\left(-\frac{Vz}{2D}\right),\;z<0 \end{array}\right.$$

The issue now is how to “work out” a solution for Equation (A17) for an originally stated dimensional distribution in an unbounded medium. Such an issue can be addressed by constructing the solution as a Fourier integral:

(A22) $$\theta \left(z,t\right)=\int_{-\infty }^{+\infty }F\left(k,t\right)\exp\left(ikz\right)dk$$

(A23) $$F\left(k,t\right)=\frac{1}{2\pi }\int_{-\infty }^{+\infty }\theta (z^{\prime },t)\exp\left(-ikz^{\prime }\right)dz\prime$$

As soon as the differential Equation (A17) is replaced, it is obtained:

(A24) $$\frac{\partial F}{\partial t}+k^{2}DF=0$$

having the following solution:

(A25) $$F\left(k,t\right)=F\left(k,0\right)\exp(-k^{2}Dt)$$

where $F\left(k,0\right)$ was extracted by supplanting the initial distribution θ into Equation (A23). As such, Equation (A22) can be stated as an integral across the whole initial distribution:

(A26) $$\theta \left(z,t\right)=\frac{1}{2\pi }\int_{-\infty }^{+\infty }\int_{-\infty }^{+\infty }\theta (z^{\prime },0)\exp\left(-k^{2}Dt\right)\exp\left[ik(z-z^{\prime })\right]dz\prime dk$$

As soon as the aforementioned integral is resolved concerning *k*, computing it for θ becomes simple, pointing towards the following:

(A27) $$\theta \left(z,t\right)=\frac{1}{{\left(4\pi Dt\right)}^{\frac{1}{2}}}\int_{-\infty }^{+\infty }\theta (z^{\prime },0)\exp\left[-\frac{{\left(z-z^{\prime }\right)}^{2}}{4Dt}\right]dz\prime$$

The solution can be concluded through substituting Equation (A21) into Equation (A27) and computing the integral. The result for $T\left(z,t\right)$ is granted as follows:

(A28) $$\Phi \left(z,t\right)=\frac{\theta_{0}}{2}\exp\left(-\frac{V^{2}t}{D}\right)+\frac{\theta_{0}}{2}\exp\left(-\frac{V^{2}t}{D}\right)\cdot \left\{erf\left[\frac{\left(z-Vt\right)}{{\left(4Dt\right)}^{\frac{1}{2}}}\right]+erf\left[\frac{\left(z+Vt\right)}{{\left(4Dt\right)}^{\frac{1}{2}}}\right]\right\}$$

where erf(x) is the Laplace function imparted by the following:

(A29) $$\mathrm{erf}\left(x\right)=\frac{1}{2\sqrt{\pi }}\int_{0}^{\infty }\exp\left(-x^{2}\right)dx$$

values of this function being tabulated.
